# Vitamin D Deficiency in Lebanese Adults: Prevalence and Predictors from a Cross-Sectional Community-Based Study

**DOI:** 10.1155/2021/3170129

**Published:** 2021-08-20

**Authors:** Asma Arabi, Nariman Chamoun, Mona P. Nasrallah, Hani M. Tamim

**Affiliations:** ^1^Department of Internal Medicine, Division of Endocrinology and Metabolism, American University of Beirut, Beirut, Lebanon; ^2^Department of Internal Medicine, Biostatistics Unit, Clinical Research Institute, American University of Beirut, Beirut, Lebanon

## Abstract

**Methods:**

A random sample of Lebanese adults residing in the Greater Beirut area was selected based on area probability and multistage cluster sampling. Data from 446 participants (68% females) with mean age 45.3 ± 15 years were used for the analyses. Participants were recruited between March and May. Serum 25-hydroxyvitamin D levels were measured using electrochemiluminescent immunoassay.

**Results:**

Vitamin D deficiency was highly prevalent whether using the cutoff of 50 nmol/L or using the more conservative cutoff of 30 nmol/L; more specifically, 71.9% and 39.1% of the study population were deficient using the above cutoffs, respectively In the bivariate analyses, gender, BMI and body fat mass, socioeconomic factors (income and education level), alcohol consumption, dietary intake of fat and of vitamin D, serum LDL-cholesterol, and serum creatinine were all associated with vitamin D status. After adjustment for multiple covariates, age, income, alcohol consumption, and serum creatinine were independent predictors of vitamin D deficiency.

**Conclusion:**

Vitamin D deficiency is highly prevalent in Lebanon. Preventive measures should target the modifiable risk factors.

## 1. Introduction

The interest in the effect of vitamin D on health has sharply increased over the past two decades, and vitamin D deficiency has been widely documented across populations [1, 2]. Although cutoff values for optimal serum 25-hydroxyvitamin D (25OHD) levels are still debatable [1], systematic reviews revealed that more than one billion people worldwide suffer from vitamin D deficiency or insufficiency, a high proportion of whom are in the Middle East [2].

Serum 25OHD is the major circulating form of vitamin D. Both 25OHD and the active metabolite 1,25-dihydroxyvitamin D, also known as calcitriol, circulate at 85% bound to vitamin D binding protein (DBP) [3]. Serum 25OHD is the best indicator of vitamin D status and depends directly on the total 25OHD and DBP levels [4]. Environmental and lifestyle factors account for the variance in serum 25OHD levels, and these include age, gender, pregnancy, latitude, sun exposure, season, pollution, clothing style, body mass index (BMI), socioeconomic status, and skin pigmentation [5, 6]. Genetic factors may also play a role [7].

There is no consensus on optimal levels of 25OHD [8, 9]. The Institute of Medicine (IOM) considers levels of 50 nmol/L and above to be sufficient. The rationale behind this cutoff point is that parathyroid hormone (PTH) starts to increase when the 25OHD level falls below 50 nmol/L, which contributes to increased bone turnover, bone fragility, and fractures [8–10]. Other studies suggest that higher serum 25OHD levels of approximately 75 to100 nmol/L may be needed to increase intestinal calcium absorption [10, 11]; hence, the Endocrine Society (ES) defines sufficiency as serum levels of 75 nmol/L and above [8]. However, several reports described the U-shaped curve between 25OHD concentration and health outcomes with levels above 100 nmol/L associated with an increased risk of falls and fractures, cancer, cardiovascular diseases, and all-cause mortality, which favors recommending a more conservative approach [12–14].

Several systematic reviews have highlighted the worldwide prevalence of vitamin D deficiency [1, 2]. Paradoxically, studies conducted in Middle Eastern countries, a sunny area, estimated that the prevalence of vitamin D insufficiency with serum levels falling below 50 nmol/L reached 90% and deficiency, with serum 25OHD levels below 30 nmol/L, up to 60% [5, 6, 15, 16].

In Lebanon, there is also a high prevalence of deficiency. A population-based study in elderly from the community in the Greater Beirut area showed a prevalence of severe vitamin D deficiency (levels falling below 25 nmol/L) in almost 55% of women and 37% of men [17]. The only study carried out on a sample of community-dwelling adults included 316 Lebanese volunteers, aged 30–50 years, recruited from rural and urban areas. It revealed that more than 75% of the sampled adults had serum levels below 30 nmol/L, with almost one-third of the study population being severely deficient (below 12 nmol/L) [18]. The study, however, dates back more than a decade and does not include young adults in the sampling frame, highlighting the need for community-based studies that include all adult age groups.

While the causes of widespread prevalence of vitamin D deficiency among the Middle Eastern population are attributed to multiple factors such as conservative clothing, time spent mostly indoors, and inadequate intake of vitamin D [18–20], other unknown factors may also be contributing to this epidemic, further highlighting the need to better characterize vitamin D status in this region.

The purpose of this study was to describe the status of vitamin D among community-dwelling adults from the Greater Beirut area in Lebanon and to determine factors associated with serum 25OHD deficiency in this population.

## 2. Methods

### 2.1. Study Design

This is a secondary analysis of deidentified data generated from the bisphenol A (BPA) study.

The original BPA study was a cross-sectional, community-based study where 501 community-dwelling adult men and women residing in the Greater Beirut area were randomly recruited between March and May 2014 [21]. All Lebanese adults over 18 years of age were eligible for inclusion. The Greater Beirut area is an urban area comprising the city of Beirut and the adjacent municipalities over the Mount Lebanon Governorate. Subjects not available at the study site and vulnerable populations were excluded, specifically pregnant women, subjects with mental disorders, and those on hemodialysis. Because the study originally assessed the effect of BPA on cardiometabolic risks, people working in a plastic or any other chemical company and therefore likely to be exposed to BPA were also excluded [21].

### 2.2. Sampling Frame

A community-based sample of Lebanese adults residing in the Greater Beirut area was selected based on area probability and multistage cluster sampling. More specifically, the districts (clusters) were selected within each district, neighborhood, and then households based on a systematic random sample according to the estimated number of buildings in the neighborhood. At the household level, a primary adult participant was selected based on the most recent day of birth. Recruitment was carried out by trained personnel. The serum 25OHD level was measured in 501 subjects enrolled in the original study; however, data on vitamin D levels and intake were available for 494 subjects only.

### 2.3. Ethical Approval

The study was approved by the Institutional Review Board Office at the American University of Beirut. Subjects were informed about the study objectives and methods, and a signed consent form was obtained from those who agreed to participate in the study.

### 2.4. Data Collection

#### 2.4.1. Anthropometrics and Demographics

Age, gender, marital status, occupation, family income, educational level, and lifestyle factors such as smoking history, alcohol intake, and medication were assessed using a validated and approved questionnaire [21]. The questionnaire also included questions on the past medical history of coronary artery disease, hypertension, dyslipidemia, diabetes mellitus, thyroid disease, cancer, fracture, and any other chronic condition [21]. Anthropometric measures including weight and height were taken using a calibrated scale; waist circumference (WC) and waist-to-hip ratio were measured using a standardized method. BMI was calculated as the ratio of weight (kilograms) to the square of height (meters). Physical activity was assessed using the short version of the International Physical Activity Questionnaire [21]. The daily dietary intake of vitamin D was assessed by calculating the reported frequency of each food item and beverage derived from the food frequency questionnaire [21]. Sitting blood pressure and heart rate were obtained twice at 10-minute intervals using a digital sphygmomanometer.

#### 2.4.2. Laboratory Assays

Serum 25-hydroxyvitamin D levels were measured using electrochemiluminescent immunoassay, with a detection limit between 7.5 and 175 nmol/L (ECLIA, Cobas e 411; Roche). Fasting plasma glucose was measured by the enzymatic method (Cobas 6000; Roche, Basel, Switzerland). The insulin level was measured by radioimmunoassay (Cisbio, Codolet, France). Serum triglycerides, high-density lipoprotein cholesterol (HDL-C), low-density lipoprotein cholesterol (LDL-C) total cholesterol, and C-reactive protein were measured using a VITROS 350 analyzer (Ortho Clinical Diagnostics, Johnson & Johnson, New Brunswick, NJ).

#### 2.4.3. Definition of Vitamin D Deficiency

There are different cutoffs to define vitamin D status:

1. The Institute of Medicine (IOM) cutoffs: deficient if <30 nmol/L; insufficient if 30–50 nmol/L; and sufficient if >50 nmol/L
2. The Endocrine Society (ES) cutoffs: deficient if <50 nmol/L; insufficient if 50–75 nmol/L; sufficient if >75 nmol/L

### 2.5. Statistical Analysis

Categorical variables were reported as frequencies and percentages, and continuous variables were reported as mean ± SD.

The mean serum vitamin D level was evaluated; and the prevalence of vitamin D deficiency was reported according to both the ES and IOM definitions [10, 13]. The remaining statistical analyses were conducted using the IOM cutoffs because of its more conservative definition of deficiency.

BMI was used in the analysis as a continuous variable and was further categorized into 4 levels: underweight (<18.5); normal (18.5–24.9); overweight (25–29.9); and obese (>30) kg/m^2^, respectively, as per the National Heart, Lung, and Blood Institute definition. Age was also categorized into 4 levels: young adult (18–35 years), middle-aged adult (35–55 years), older adults (55–65 years), and elderly (>65 years). The chi-square test or ANOVA was used to assess the association at a bivariate level between predictors and vitamin D levels as per IOM definitions. Gamma measure was calculated for ordinal variables. A logistic model was built to adjust for multiple predictors. The model was built using the all enter method with vitamin D status being the outcome (deficient versus insufficient and sufficient combined). The predictors included all variables that showed statistical association with 25OHD levels in bivariate analyses, which were thought to be associated as predictors, rather than as outcomes; these were gender, education, income, alcohol consumption, and serum creatinine. Although age was not associated with vitamin D status in bivariate analyses, we entered it in the model because age was widely reported as a predictor of serum 25OHD level in the literature.

*p* value ≤ 0.05 was considered statistically significant. All statistical analyses were completed using Statistical Package for Social Sciences (SPSS, version 24; IBM Corp, Armonk, NY).

## 3. Results

### 3.1. Demographics and Anthropometric Characteristics

Out of 494 participants who had vitamin D levels available, 28 (5.7%) were taking vitamin D supplements. Analysis for the prevalence of deficiency did not differ whether including or excluding those on vitamin D supplementation. Therefore, we report the remainder of the analysis using only the sample with 466 participants (after excluding those taking supplements). The mean age was 45.3 ± 15 years, with 41.8% being middle-aged adults (ranging between 35 and 55 years) and 9.0% being elderly (age 65 years of age). The majority of study participants were females (63.0%), married (68.5%), and 36.0% received no or only primary education. More than half of the study sample population were cigarette or water-pipe smokers (57.3%), 84.4% were physically active, and 74.7% never drank alcohol. The mean BMI was 29.2 ± 5.8 kg/m^2^ with 41% of participants being obese and 34% being overweight. Reported chronic conditions included diabetes (13.7%), dyslipidemia (22.9%), and hypertension (55.5%).

### 3.2. Prevalence of Vitamin D Deficiency

The mean 25OHD level in the overall population was 40.4 ± 23.2 nmol/L (37.5 ± 25.8 in men and 45.3 ± 16.4 in women, *p* < 0.0001). The prevalence of vitamin D deficiency and insufficiency according to IOM and ES classifications is shown in Table 1. There was a large difference in this prevalence between IOM and ES. Using IOM cutoffs, more than one-third of the study population were deficient (39.1%); this proportion almost doubled when using the ES cutoffs with 71.9% of the study population being deficient and only 8.4% had sufficient levels.

### 3.3. Relationship between Vitamin D Status and Potential Predictors

There was no association between age and vitamin D status, with mean ± SD age of 44.3 ± 14 versus 44.4 ± 15.7 and 45.6 ± 16.3 in the deficient, insufficient, and sufficient groups, respectively. The youngest group (18–35 years) represented almost one-third of each vitamin D category (Table 2).

Gender was significantly associated with vitamin D deficiency, whereby 83.5% of the deficient group were females (*p* < 0.001). Moreover, using the IOM classification, 51.5% of women had deficient levels and 25.4% only had sufficient levels. In men, these percentages were 17.5% and 32.7%, respectively (*p* < 0.001 for difference between genders) (Table 1).

Socioeconomic status parameters such as educational level and income were inversely associated with vitamin D status. Indeed, the majority of participants who received primary education or less were deficient or insufficient (75.6%), and only 24.4% were sufficient (Table 2). Moreover, the proportion of subjects who were vitamin D deficient increased from 24% in those who achieved university education to 44.6% in those who did not receive primary education (*p*=0.04) (Table 2). In addition, income was inversely correlated with vitamin D levels, with 44.1% of subjects who reported monthly income below 600 US dollars being deficient versus 25.7% of those who reported a monthly income above 2000 US dollars (*p*=0.02) (Table 2).

Alcohol consumption was inversely associated with vitamin D sufficiency status where those who reported never drinking alcohol were more likely to be deficient than current consumers (47.2% versus 13.5%) (Table 2). There was no association between physical activity or marital status and vitamin D status.

Deficient subjects were more likely to be obese than sufficient ones (46.3% versus 35.9%, *p*=0.03) with BMI being higher in the deficient group (30.1 ± 6.1 kg/m^2^) than insufficient (28.5 ± 5.3 kg/m^2^) and sufficient (28.1 ± 5.3 kg/m^2^) groups, *p*=0.02. A similar trend was also noted for percent body fat (Table 3). In addition, mean fat intake and daily dietary vitamin D intake were significantly lower in the vitamin-D-deficient group than other groups (*p*=0.001). Mean serum creatinine was lower in the deficient subjects (*p* < 0.001).

### 3.4. Independent Predictors

Age, alcohol consumption, income, and serum creatinine were significant independent predictors of vitamin D status (deficient versus insufficient/sufficient combined). BMI showed borderline significance (Table 4).

### 3.5. Relationship between Vitamin D Status and Potential Health Outcomes

The LDL-cholesterol levels were higher in deficient (115.1 ± 42.8 mg/dL) than in insufficient (107.7 ± 34.2 mg/dL) and sufficient subjects (100.6 ± 32.6 mg/dL), *p*=0.004. Diastolic blood pressure was slightly higher in the insufficient group than the two other groups, *p*=0.04. However, there was no association between the prevalence of hypertension or dyslipidemia and vitamin D status. Similarly, there was no association with the prevalence of diabetes mellitus, thyroid disease, cancer, or reported fractures (Table 3).

## 4. Discussion

This study showed that vitamin D deficiency is still highly prevalent among community-dwelling adults in Lebanon. The likelihood of being deficient increased with low socioeconomic status. According to the ES classification, around two-thirds of the participants were vitamin D deficient, and even using the more conservative cutoff of the IOM, the prevalence was 39.1%, which remains quite elevated for a country located at the latitude of 33° North with around 300 sunny days a year. This high prevalence of vitamin D deficiency is in line with findings from previous reports from Lebanon and the Middle Eastern regions [1, 5, 6].

Although the prevalence of vitamin D deficiency is high, the 39.1% prevalence using IOM threshold was lower than the one reported by Ghannagé-Yared et al. in a similar age group population (30 to 50 years) from both rural and urban areas from Lebanon in the year 2000, where 75% of the study population were deficient using the same threshold [18]. Furthermore, the mean 25OHD levels of 40.4 ± 23.2 nmol/L in the current study were higher compared with those reported by Gannage-Yared et al. where the reported mean values were 25 ± 17.5 nmol/L [18] and compared with those reported by our group in elderly population (65–85 years) residing in the Greater Beirut area in 2006, whereby the mean serum vitamin D was 27.5 ± 12.5 nmol/L [6]. This may reflect improvement over time in the vitamin D status in our population. Improvement in vitamin D status happens if people take vitamin D from fortified food or from supplements. In the current study, subjects taking supplements were excluded; however, their number was very small (28 participants only), and even when we conducted the analyses in the overall group including those on supplements, this did not affect the overall prevalence. Possible reasons for the improved levels could be higher exposure to sunlight as a result of increased awareness about the role of sunlight in vitamin D synthesis and human health, leading to changes in lifestyle such as outdoor activities. Another possible reason for improvement in vitamin D status over time is the higher dietary intake. In this study, intake from fortified food was different between sufficient and deficient groups (108 IU versus 66.8 IU daily), but the overall daily intake in all groups remains far below the IOM-recommended daily requirements of 600–800 IU to reach a sufficient 25OHD level of 50 nmol/L. Evidence from both observational studies and randomized controlled trials suggest that sufficiency levels are derived from a combination of diet, fortified foods, and supplementation, whereby each 100 IU of vitamin D daily can raise serum concentrations by 1 ng/mL after 2 to 3 months [22]. In the current study, daily dietary intake was lower in the deficient than sufficient group and lower than intake reported in other studies worldwide [23, 24]. Data from the NHANES cohort (2004–2006) for instance revealed that the mean dietary intake of vitamin D among adults is 216 ± 1.2 IU/day [23]. The study from a German cohort revealed that the mean dietary intake of vitamin D among the German elderly is equivalent to 124 ± 16 IU/day, with higher mean levels reported among males [24].

Another explanation for the difference of vitamin D status over time may be related to the different assays used for the measurement of vitamin D level. The current study used electrochemiluminescent immunoassay, whereas Ghannagé-Yared et al. used radioimmunoassay [18]. Difference in the measured 25OHD levels using different assays were indeed reported [25]; however, Connell et al. compared the performance of ECLIA and RIA methods with the gold standard liquid chromatography tandem mass spectrometry (LC-MS/MS) assay in two different sets of population and concluded that ECLIA underestimates the levels compared with other methods [26]. Seasonal variability is another possible cause underlying the difference in levels since the samples in the Rosecrans and Dohnal study were collected between January and April, whereas in the current study samples were collected in spring (March to May). Indeed, seasonal variability in 25OHD levels has been reported in population-based studies, across age groups and different latitudes [27]. As an example, in Turkey, in a meta-analysis of 40 studies published between 2000 and 2017, Alpdemir and Alpdemir reported an overall prevalence of deficiency of 63.5% using the IOM cutoff, across all adult age groups. The prevalence varied widely across different cities according to the season and the latitude of each city. For the cities of Trabzon (latitude 41°) and Izmir (latitude 27°), the prevalence of deficiency was 93% and 59% at the end of winter, respectively. And it dropped to 25% and 7%, respectively, by the end of summer [28]. Seasonal variability of vitamin D levels was also reflected in a population-based study from Athens, Greece, on postmenopausal women with osteoporosis with deficiency being 76.5% in March and dropping to 38.1% by August [29]. Finally, the lower level of deficiency in our study may reflect a true increase in vitamin D levels. Indeed, in a recent paper, Saad et al. analyzed data from 151,705 laboratory tests performed at out academic institution from 2009 to 2016, where cross calibration formulas were used to convert measured 25OHD levels to LC-MS/MS equivalents. In that study, a real increase of 3 nmol/liter/year in serum 25OHD levels in adults aged 19–64 years was observed. Vitamin D deficiency was still prevalent in the entire population with 23% being deficient using cutoffs on 30 nmol/L across all years [30].

A higher prevalence of vitamin D deficiency was similarly found in other countries from the region. In a population-based study in Bahrain [31], only 13.6% of the community-dwelling study population aged 15–65 years, of both genders, had levels above 50 nmol/L using the LC-MS/MS assay [31]. In that study, samples were collected all year round and the proportion of deficient subjects was significantly higher during October to March (69.2%) than April to September (12.5%). Another population-based study conducted in Jordan in 2010, including 2032 young women aged 15 to 49 years from both rural and urban areas, and using LC-MS/MS assay, showed that all women were deficient or insufficient, with 60.3% having levels below 30 nmol/L [32]. Clothing style may also partially account for the gender differences observed from other community-based studies among adults in Bahrain, Saudi Arabia, Syria, and the United Arab Emirates [31, 33–36] where women had lower levels than men. In our study, mean 25OHD levels were lower in women than men. However, the effect of gender was not significant after adjustment for other covariates.

On the other hand, age was a weak but significant positive predictor of deficiency in our study, as expected and supported by the literature [5].

We found an inverse association between BMI and serum 25OHD concentrations. Our findings are consistent with the literature from the same age group in the region [31–36], whereby BMI and obesity are suggested as determinants of vitamin D deficiency, with a negative correlation between BMI and mean serum vitamin D. Vitamin D is a fat-soluble vitamin, and the bioavailability of vitamin D decreases in obesity because of its storage in body fat. Findings from the Global Burden of Disease study showed a high prevalence of obesity in the Middle East [37]. This high prevalence of obesity may be a contributing factor to the low vitamin D levels observed in the region and shed the light on the need to consider body mass index and fat levels in daily recommended dose of vitamin D supplementation in this area.

Besides age, gender, and BMI, other independent predictors of vitamin D status were socioeconomic factors such as educational level and income. These were inversely associated with vitamin D status and may reflect health inequities, where poor people cannot afford fortified food, or reflects lower awareness about the importance of outdoor activities in the less privileged and less educated people. Moreover, obesity is more common in people from lower socioeconomic status, thus another possible explanation of lower vitamin D levels in this group.

Alcohol consumption was another independent predictor of vitamin D status with a positive association showing higher vitamin D levels in alcohol consumers versus nonconsumers. Inconsistent results about the association between alcohol consumption and vitamin D status were reported from Western countries. Tardelli et al. [38] reviewed this association in 49 papers and found that alcohol consumption was positively associated with vitamin D status in 15 studies, whereas the association was negative in 18 studies, and no association was found in 16 studies. This difference in the results could be due to many factors, such as the country latitude, social habits, and amount of alcohol consumed. For example, in studies conducted in high-latitude countries with cold weather, negative association may be explained by higher alcohol consumption levels. In the Lebanese community, alcohol consumption in general is mild to moderate. Another speculative mechanism is that alcohol consumption suppresses parathyroid hormone secretion, thereby decreasing the conversion of serum 25OHD into 1,25OH2D and consequently increasing 25OHD levels [39]. Additional factors accounting for the different study findings could be malabsorption, poor dietary intake, or direct effect on vitamin D metabolism [38].

Serum creatinine was positively associated with vitamin D levels where the sufficient group had the highest serum creatinine. An explanation for the higher creatinine in sufficient subjects may be the fact that vitamin D is fat soluble, and therefore those with less body fat, and consequently higher lean mass, are expected to have higher 25OHD levels. We did not adjust for fat or lean mass in the multivariate analyses because we adjusted for BMI and the effect of BMI on 25OHD level is, at least in part, mediated by these two variables.

Interestingly, vitamin D itself may affect creatinine generation by muscle and therefore serum creatinine levels without affecting kidney function. The latter was demonstrated in an elegant study by Agarwal et al., on 16 patients with chronic kidney disease, whereby an infusion of paricalcitol (a vitamin D receptor activator) resulted in an increase in serum creatinine, without change in the glomerular filtration rate [40]. However, kidney function was normal in our study population, making the former association more plausible.

The serum LDL-C level was negatively associated with mean serum vitamin D; such correlation has been reported in other studies in the Middle East [41, 42]. Obesity may be a mediator to both conditions, but a physiologic explanation to this inverse association is that vitamin D and cholesterol share the same metabolic pathway.

This study has some limitations. The cross-sectional design does not allow to confirm a causal relationship between the predictors and vitamin D status at the time of participation. In addition, the study lacks data on clothing style and sunscreen use, as well as information on outdoor activities, all of which are related to sunlight exposure, a major predictor of vitamin D. This is not a population-based study; however, it is a community-based study using multistage cluster sampling, enrolling a large number of participants from a geographic area that represents 30% of the Lebanese population.

In conclusion, vitamin D deficiency is still highly prevalent in the Lebanese population, even when using the conservative classification of the IOM. Age, gender, socioeconomic status, serum creatinine, and BMI are main predictors. Preventive measures should target modifiable risk factors.

## Figures and Tables

**Table 1:** Prevalence of vitamin D deficiency based on the Institute of Medicine and Endocrine Society definitions.

	Overall		Men		Women	
25OHD	40.4 ± 23.21		37.50 ± 25.88		45.32 ± 16.46	
Mean ± SD (nmol/L)								
	Institute of Medicine^*∗*^	Endocrine Society^*∗∗*^	Institute of Medicine^*∗*^	Endocrine Society^*∗∗*^	Institute of Medicine^*∗*^	Endocrine Society^*∗∗*^
	*n* (%)	*n* (%)	*n* (%)	*n* (%)	*n* (%)	*n* (%)
Deficient	182 (39.1%)	335 (71.9%)	30 (17.5%)	115 (67.3%)	152 (51.5%)	220 (74.6%)
Insufficient	153 (32.8%)	92 (19.7%)	85 (49.7%)	44 (25.7%)	68 (23.1%)	48 (16.3%)
Sufficient	131 (28.1%)	39 (8.4%)	56 (32.7%)	12 (7.0%)	75 (25.4%)	27 (9.2%)

^*∗*^Deficient (<30 nmol/L); insufficient (30–50 nmol/L); sufficient (>50 nmol/L). ^*∗∗*^Deficient (<50 nmol/L); insufficient (50–75 nmol/L); sufficient (>75 nmol/L).

**Table 2:** Bivariate association of 25OHD level categorized based on the Institute of Medicine definition with sociodemographic and lifestyle factors (*N* = 466).

		Deficient^*∗*^	Insufficient†	Sufficient	*p* value
*Sociodemographic characteristics*					
Age (mean ± SD)		44.3 ± 14.0	44.4 ± 15.7	45.6 ± 16.3	0.41
Age categorized	18–35	51 (36.4%)	47 (33.6%)	42 (30.0%)	0.16
	35–55	84 (43.1%)	63 (32.3%)	48 (24.6%)	
	55–65	34 (39.1%)	32 (36.8%)	21 (24.1%)	
	>65	12 (28.6%)	11 (26.2%)	19 (45.2%)	
Gender	Female	152 (51.5%)	68 (23.1%)	75 (25.4%)	**<0.001**
	Male	30 (17.5%)	85 (49.7%)	56 (32.7%)	
Marital status	Single	85 (49.7%)	38 (40.0%)	30 (31.6%)	0.11
	Married	56 (32.7%)	91 (29.6%)	84 (27.4%)	
	Other	23 (35.9%)	24 (37.5%)	17 (26.6%)	
Education	No/primary	75 (44.6%)	52 (31.0%)	41 (24.4%)	**0.04**
	Intermediate	55 (44.4%)	39 (31.5%)	30 (24.2%)	
	Secondary	37 (30.8%)	40 (33.3%)	43 (35.8%)	
	University	12 (24.0%)	21 (42.0%)	17 (34.0%)	
Income	<600$	60 (44.1%)	49 (36.0%)	27 (19.9%)	**0.02**
	600–< 2000$	94 (38.1%)	72 (29.1%)	81 (32.8%)	
	>2000$	9 (25.7%)	16 (45.7%)	10 (28.6%)	

*Lifestyle characteristics*					
Cigarette smoking	Never	88 (40.0%)	64 (29.1%)	68 (30.9%)	0.18
	Current	81 (40.7%)	71 (35.7%)	47 (23.6%)	
	Exsmoker	13 (27.7%)	18 (38.3%)	16 (34.0%)	
Water-pipe smoking	Never	112 (39.2%)	98 (34.3%)	76 (26.6%)	0.11
	Current	60 (44.1%)	38 (27.9%)	38 (27.9%)	
	Exsmoker	10 (22.7%)	17 (38.6%)	17 (38.6%)	
Alcohol	Never	163 (47.2%)	97 (28.1%)	85 (24.6%)	**<0.001**
	Current	12 (13.5%)	44 (49.4%)	33 (37.1%)	
	Exdrinker	7 (21.9%)	12 (37.5%)	13 (40.6%)	
Physical activity	Any PA	155 (39.3%)	128 (32.5%)	111 (28.2%)	0.92

^*∗*^Deficient (<30 nmol/L); insufficient (30–50 nmol/L); sufficient (>50 nmol/L). ^†^Deficient (<50 nmol/L); insufficient (50–75 nmol/L); sufficient (>75 nmol/L).

**Table 3:** Bivariate association of vitamin D level categorized based on the Institute of Medicine definition with health conditions, medications, lab results, and daily dietary intake of vitamin D (*N* = 466).

	Deficient^*∗*^	Insufficient^†^	Sufficient	*p* value
	Mean ± SD	Mean ± SD	Mean ± SD	
SBP^‡^, mmHg	119.7 ± 19.1	124.4 ± 19.9	120.9 ± 18.6	0.06
DBP^‡^, mmHg	73.7 ± 10.0	76.9 ± 10.0	73.6 ± 9.6	**0.04**
Hypertension, *n* (%)	88 (48.4%)	102 (66.7%)	69 (53.1%)	0.20
Diabetes, *n* (%)	30 (16.5%)	17 (11.1%)	17 (13.0%)	0.34
Thyroid disease, *n* (%)	42 (23.1%)	18 (11.8%)	28 (21.4%)	0.47
Dyslipidemia, *n* (%)	37 (20.3%)	39 (25.5%)	31 (23.7%)	0.52
Fracture, *n* (%)	47 (25.8%)	46 (30.1%)	36 (27.5%)	0.66
Cancer, *n* (%)	1 (0.5%)	2 (1.3%)	4 (3.1%)	0.11
BMI, kg/m^2^	30.1 ± 6.1	28.5 ± 5.3	28.1 ± 5.3	**0.02**
Obesity, *n* (%)	84 (46.2%)	61 (39.9%)	47 (35.9%)	**0.03**
Body fat, kg	31.2 ± 11.4	27.0 ± 10.9	26.2 ± 10.7	**<0.001**
Muscle mass, kg	25.9 ± 6.4	27.2 ± 6.6	25.8 ± 6.3	0.11
Waist-to-hip ratio	0.97 ± 0.08	0.97 ± 0.07	0.96 ± 0.1	0.58
Waist circumference, cm	96.3 ± 17.0	96.4 ± 14.3	93.2 ± 13.8	0.14
Glucose, mg/dL	113.5 ± 48.0	107.6 ± 29.5	112.6 ± 48.8	0.42
Serum creatinine, mg/dL	0.67 ± 0.16	0.82 ± 0.25	0.80 ± 0.22	**<0.001**
Cholesterol, mg/dL	191.8 ± 49.7	184.5 ± 39.8	178.9 ± 38.9	**0.035**
Triglyceride, mg/dL	145.8 ± 136.2	141.2 ± 73.6	137.9 ± 83.2	0.80
HDL, mg/dL	49.5 ± 14.6	49.0 ± 15.3	50.2 ± 14.5	0.77
LDL, mg/dL	115.1 ± 42.8	107.7 ± 34.2	100.6 ± 32.6	**0.004**
Sum of fat, g/day	120.7 ± 73.7	155.5 ± 90.3	142.1 ± 91.4	**0.001**
Sum of vitamin D, IU/day	66.8 ± 60.7	103.2 ± 88.3	108.0 ± 99.9	**0.001**

^*∗*^Deficient (<30 nmol/L); insufficient (30–50 nmol/L); sufficient (>50 nmol/L). ^†^Deficient (<50 nmol/L); insufficient (50–75 nmol/L); sufficient (>75 nmol/L). ^‡^SBP: systolic blood pressure, DBP: diastolic blood pressure.

**Table 4:** Multivariate model for predictors of vitamin D status, using the Institute of Medicine classification (*N* = 466).

Vitamin D status based on IOM criteria (sufficient/insufficient^†^ vs. deficient^*∗*^)		
	OR (95% CI)	*p* value
Age	1.02 (1.00–1.04)	**0.03**
Gender: female	1.35 (0.75–2.45)	0.32
BMI	0.96 (0.92–1.00)	0.054

*Education*		
(i) Intermediate	0.97 (0.55–1.72)	0.91
(ii) Secondary/technical	1.56 (0.88–2.77)	0.13
(iii) University education	1.54 (0.71–3.33)	0.27

*Income*		
600–<2000$	1.95 (1.20–3.17)	**0.007**
>2000$	1.11 (0.44–2.78)	0.83

Alcohol: current	1.85 (1.08–3.17)	**0.03**
Serum creatinine	3.31 (1.06–10.33)	**0.04**

Variables included in the model are age, gender (reference: male), education (reference: no/primary), income (reference: <600$), alcohol (reference: never), BMI, and serum creatinine. ^*∗*^Deficient (<30 nmol/L); insufficient (30–50 nmol/L); sufficient (>50 nmol/L). ^†^Deficient (<50 nmol/L); insufficient (50–75 nmol/L); sufficient (>75 nmol/L).

## Data Availability

All data presented in the manuscript are available upon request.
